# A Community Study on Sleep Characteristics and Anxiety Symptoms in Children with Dyslexia

**DOI:** 10.3390/brainsci15070711

**Published:** 2025-07-01

**Authors:** Katrin Jeffcock, Dagmara Dimitriou

**Affiliations:** Sleep Education and Research Laboratory, UCL Institute of Education, University College London, London WC1E 6BT, UK; katrin.jeffcock.18@ucl.ac.uk

**Keywords:** sleep, dyslexia, anxiety

## Abstract

**Objectives:** Sleep serves a crucial role in the optimal development of cognitive, emotional, and physical domains. Sleep disturbances and disorders have been reported to frequently occur in many neurodevelopmental conditions, including ADHD and Autism Spectrum Disorder. The connection between dyslexia and sleep, however, is sparsely explored. This community study aimed to enhance knowledge about sleep disturbances in children with dyslexia and explore the potential impact of anxiety. **Method:** The parents of 160 children aged 7–13 years old with a primary diagnosis of dyslexia completed the Children’s Sleep Habits Questionnaire (CSHQ) and the Spence Children’s Anxiety Scale (SCAS). **Results:** Sixty-six percent of the children showed pathological levels of sleep disturbances, with clinical scores observed in the subscales of Sleep Onset Delay, Sleep Anxiety, and Daytime Sleepiness. Overall, sleep and anxiety were correlated, but anxiety levels were not elevated and not correlated with Sleep Onset Delay. **Conclusions:** The current results suggest that the majority of children with dyslexia suffer from sleep disturbances, such as delayed sleep onset and shorter sleep durations, irrespective of the scores given on the anxiety scale. Given the importance of sleep for optimal development, there is an alarming need for more studies to be carried out to explore additional factors that interact with healthy sleep to develop sleep interventions.

## 1. Introduction

Dyslexia is a neurodevelopmental condition (NDC) affecting accurate and fluent word reading and spelling, with an estimated global prevalence rate of around 7% [1]. A complex interplay between multiple genetic and environmental risk factors has been associated with dyslexia; the influence of such factors on neural systems and cognitive processes ultimately leads to the behavioral symptoms observed in dyslexia [2]. In recent years, a significant amount of research has examined dyslexia within the broader classification of specific learning disorders (SLDs) with a focus on the cognitive, genetic, and neural foundations of dyslexia, particularly its association with reading fluency and spelling. Despite this, some areas remain to be examined, including, in particular, the relationship between dyslexia and sleep behavior, to enhance our understanding of dyslexia and develop more effective interventions.

This lack of research is surprising, given that sleep disturbances are included in the diagnostic criteria for many neurodevelopmental disorders [3]. Sleep is widely recognized to serve an essential role in cognitive development [4,5,6,7], and sleep disturbances have been found to lead to reduced cognitive functioning, behavioral problems, and reduced quality of life. Sleep disturbances in NDCs can arise for a variety of reasons, and early identification is important to identify the most effective treatment to improve the quality of life of an individual [8]. Indeed, sleep difficulties have been extensively reported in children with NDCs [9,10], such as autism [11,12,13], Williams Syndrome [14], and Down Syndrome [15]. A striking 10,632 publications focused on ADHD and sleep between 1970 and 2018 [16]. In contrast, fewer than 20 studies examined the relationship between dyslexia and sleep disturbances [17]. Despite the limited number of studies, the evidence seems to point to a greater presence of sleep disorders in children with dyslexia.

The first large questionnaire-based study exploring sleep disturbances in children with dyslexia reported total elevated scores on the Sleep Disturbance Scale for Children (SDSC) [18], suggesting significant sleep disturbances in dyslexia. Specifically, 44.2% of children with dyslexia scored above the norm on the “Disorders in Initiating and Maintaining Sleep” subscale, compared to only 8.3% of typically developing (TD) individuals who scored above the cut-off mark [19]. Similarly, in a recent community sample study using data from sleep services, children with a primary diagnosis of learning difficulties/dyslexia/dyspraxia showed clinical scores for sleep disturbances [10]. Additionally, in a study exploring sleep in children with ADHD and SLD, 24% of children diagnosed exclusively with SLD showed Disorders in Initiating and Maintaining Sleep at a clinical level [20].

Alterations in the microstructure or functionality of sleep in children with dyslexia have also been reported; however, studies using objective electroencephalography (EEG) are limited and have small sample sizes (*n* < 30). Sleep architecture was found to be altered in children with reading disabilities [21], and several studies showed unusually long phases of the slow-wave sleep stage and an increase in sleep spindle density, which were interpreted as signs of enhanced cortico-cortical coupling that may support the compensatory system to facilitate learning and consolidation processes in children with dyslexia [22,23,24]. In contrast, other studies examining sleep-related memory consolidation suggested that sleep difficulties may exacerbate learning difficulties related to dyslexia [25,26]. Psychosocial stress in childhood and adolescence caused by performance difficulties associated with dyslexia can lead, over time, to adaptations in the HPA axis and disruptions in regulatory processes. It has been proposed that dyslexia may represent a positive evolutionary adaptation to challenges related to stress homeostasis [27] with disturbing effects on the circadian rhythm. Similar to findings from a study examining cortisol and melatonin levels in children with Williams Syndrome [28], a study carried out by Huang and colleagues [29] reported that individuals with dyslexia also exhibit lower levels of hormonal variations and a reduced diurnal rhythm. Similarly, Espin and colleagues [30] reported reduced cortisol levels in response to a stress test in the dyslexia group compared to controls. This suggests that dyslexia is also associated with disruptions in the normal patterns of hormonal regulation and circadian rhythms. Such disturbances may contribute to broader cognitive and emotional challenges, such as the high anxiety levels experienced by children with dyslexia.

### 1.1. Dyslexia and Anxiety

It has been hypothesized that sleep difficulties in children with dyslexia could be related to increased anxiety, which is often an indicator of and comorbid with developmental disorders [25,31]. A complex, bi-directional relationship has been suggested between anxiety and sleep, where anxiety has previously been shown to be bidirectionally related to insomnia [32,33]. In TD children, phobia of school has been found to have the strongest association with sleep problems [34]. Children with dyslexia, compared to TD children, have been found to show similar anxiety profiles but higher generalized anxiety levels [35] and comorbid anxiety conditions [36]. Prevalence rates are as high as 69% [37]. Stronger effects have been found in girls [38], and anxiety has been found to be specifically related to reading measures [39]. The proportionally higher need for children with dyslexia to have their parents in the room at bedtime [10] might be an indication of increased anxiety. Similarly, during the recent pandemic period, the parents of children with dyslexia reported that almost 20% were unable to sleep alone, in contrast to children with autism or intellectual disabilities, where the rates were slightly lower [40]. Although not all studies found a direct link between dyslexia and anxiety symptoms [41], it is likely that anxiety symptoms may be associated with sleep in children with dyslexia.

### 1.2. The Current Study

Given that dyslexia is a common neurodevelopmental condition, and sleep serves an essential role in cognitive [42,43,44] language development [45], it is essential to deepen our understanding of the interaction between sleep and dyslexia. Furthermore, considering the well-established relationship between anxiety and sleep [46] and the high comorbidity of anxiety in children with dyslexia [47], exploring how anxiety might impact sleep in individuals with dyslexia is particularly important. Understanding these interactions can provide valuable insights into the broader cognitive and emotional challenges faced by individuals with dyslexia and inform more effective interventions in educational settings and healthcare.

The present study is the first large community study in the UK examining the characteristics of sleep in children with a primary diagnosis of dyslexia and considering anxiety scores as a potential mediator of sleep quality. Firstly, this study will investigate the prevalence of sleep disturbances and sleep duration in children with dyslexia. Based on findings from previous research, we anticipate that a significant proportion of children with dyslexia will score within the clinical ranges on the sleep questionnaires.

Secondly, considering that dyslexia can trigger school-related anxiety and that anxiety can impact sleep quality, we expect to find a significant association between anxiety symptoms and sleep quality in this population.

## 2. Method

### 2.1. Participants

A total of 789 carers or parents started filling in the questionnaire within 6 weeks; 509 parents gave valid consent, of which 58 were excluded for not living in the UK, and 449 participants were within the age limit of 7–13 years (*M* = 9.94, *SD* = 1.68, 57% male). In total, 74% of participants (*n* = 333) reported having a primary diagnosis of a learning disability, dyslexia, or dysgraphia (“dyslexia” in further analysis), 7% (*n* = 33) reported a co-occurring diagnosis, and 11% (*n* = 49) reported a suspected diagnosis. Forty-six percent of the participants (*n* = 208) had a diagnosis or suspicion of at least one other co-occurring condition; 14.9% of all participants (*n* = 67) took at least one of the listed medications for study exclusion. To gain a clearer understanding of any condition-specific outcomes, children with other diagnoses and other suspected diagnoses were excluded, and only children with a primary diagnosis of dyslexia were included in the analysis. To eliminate any possible influence of medication on sleep behavior, 18 children reporting the intake of medication were excluded. The final dataset for analysis consisted of 160 children with a mean age of 9.78 years (*SD* = 1.68, 57.2% male), a primary diagnosis of dyslexia only, living in the UK, and not taking any medication. Missing data in the sleep and anxiety questionnaires were pairwise deleted, resulting in a different sample size used in the analysis for each variable.

### 2.2. Recruitment

An invitation to caregivers of children with dyslexia aged 7–13 was distributed online in the UK dyslexia support community and via branches of the British Dyslexia Association. Ethical approval was obtained from the UCL IOE Ethics Committee for the research protocol (Data Protection number: D6364106/2022/01/02). All data management was in compliance with GDRP guidelines. Participants were provided with an online link or QR code to complete the questionnaire online via Qualtrics XM software (Qualtrics, Provo, UT, USA).

### 2.3. Materials

The Children’s Sleep Habits Questionnaire (CSHQ) [48] is a 45-item caregiver report covering major clinical presentations of childhood sleep problems, of which 33 items grouped into 8 subscales were employed (Bedtime Resistance, Sleep Onset Delay, Sleep Duration, Sleep Anxiety, Night Wakings, Parasomnias, Sleep Disordered Breathing, and Daytime Sleepiness). Items were reported by caregivers on a 3-point Likert scale (with 1 being rarely and 3 being usually), where higher scores indicated greater sleep disturbances. The remaining 7 items with follow-up questions pertaining to sleep behavior were omitted to reduce the load on participants [49]. The CSHQ has high internal validity, and Cronbach’s Alpha for the total CSHQ of the current sample was acceptable at 0.74 [50]. A total above the cut-off score of 41 indicated clinical levels of sleep disturbance [48].

The Spence Children’s Anxiety Scale (SCAS) [50] is a 38-item caregiver report measuring anxiety symptoms in children aged 6–16 and is used in the measurement of symptoms of DSM-V anxiety disorders in both mainstream and non-mainstream settings. Items are reported by caregivers on a 4-point Likert scale (with 0 being never and 3 being always) and grouped into 6 subscales (separation anxiety, social phobia, obsessive–compulsive disorder, panic disorder/agoraphobia, personal injury fears, and generalized anxiety). The SCAS has high internal validity, and Cronbach’s Alpha score of the current sample was 0.86 [51,52,53].

Scores above the population norms can be interpreted as indicative of “elevated levels of anxiety”. The population norms are divided by gender and age. For boys, total SCAS scores of 40 and above were indicative of an “elevated level of anxiety” for ages 8–11 years old, and scores of 33 and above were indicative of an “elevated level of anxiety” for ages 12–15 years old. For girls, total SCAS scores of 50 and above were indicative of an “elevated level of anxiety” for ages 8–11 years old, and scores of 40 and above were indicative of an “elevated level of anxiety” for ages 12–15 years old.

### 2.4. Analysis Approach

Data were analyzed using IBM SPSS (SPSS Inc., Chicago, IL, USA, Version 28). Considering the sample size, normal distribution was assumed, and parametric tests were used for improved statistical power [54]. Independent sample t-tests were performed to check for gender differences in the CSHQ and SCAS total scores and subscale scores. Regression analysis was conducted to check for the effects of age on the CSHQ and SCAS total scores. Pearson correlations were used to assess the associations between CSHQ and SCAS total scores and subscales and the average hours of sleep gained per night. The descriptive statistics of the CSHQ and SCAS were calculated. An additional subgroup was formed for children who scored within the clinical range based on the CSHQ total score (“Poor Sleepers”), enabling the comparison of sleep and anxiety scores between children with dyslexia and sleep problems and clarifying the type of sleep problems these children with dyslexia face. The average number of hours of sleep gained per night was calculated, and a subgroup of “<9 h sleepers” was created for children with dyslexia who slept less than the recommended 9 h per night [55].

## 3. Results

### 3.1. Correlation Analysis

Pearson correlation coefficients were calculated for all the study variables from CHSQ and SCAS (Table 1).

### 3.2. Age and Gender Effects

Independent t-tests showed no significant differences between genders on the total CSHQ and CSHQ subscales and the total SCAS subscales. Hence, the sample was analyzed together for subsequent statistical analyses. Age was also not a significant predictor of the total CSHQ and SCAS scores; this was only a significant predictor of the (i) CSHQ Sleep Anxiety subscale, *F*(1, 126) = 4.34, *p* = 0.039 and (ii) SCAS physical injury subscale, *F*(1, 126) = 4.12, *p* = 0.045.

### 3.3. Sleep Characteristics Based on the CSHQ Parental Scores

The mean total CSHQ score was 46.94, which is above the clinical cut-off point of 41, and the subscale Sleep Onset Delay (SOD) was 1.85, above the clinical cut-off of 1.80 [48]. All subscales of the total CSHQ were significantly correlated with the total CSHQ for the sample (Table 1). The descriptive statistics of total CSHQ scores are summarized in Table 2, and subscale values for TD children are presented for comparison from the TD sample from Owens [48] as the gold standard for TD without other potentially interfering conditions.

In total, 66% of all children with dyslexia scored above the clinical cut-off score (*n* = 76, *M* = 51.86, *SD* = 7.17) (“Poor Sleepers”). The descriptive statistics of the CSHQ scores in this group are summarized in Table 3. A chi-square test found that clinical sleep characteristics were significantly more likely in this sample of children with dyslexia, χ^2^(1) = 18.24, *p* < 0.001.

### 3.4. Average Sleep Duration

**Figure 1:**
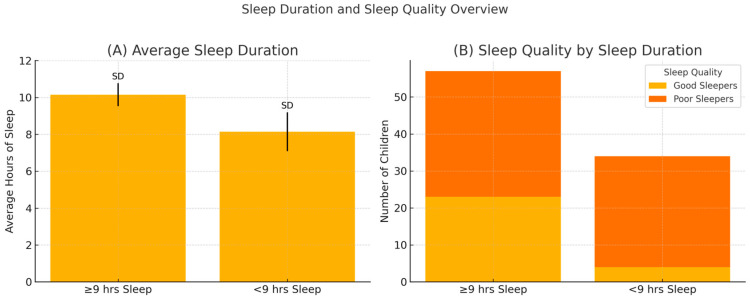
Comparison of average sleep duration and sleep quality among children who sleep less than versus at least 9 h per night. (**A**) Mean total sleep duration (in hours) with standard deviations for each group. (**B**) Distribution of sleep quality (good vs. poor sleepers) within each sleep duration group.

The average total hours of sleep per night was significantly correlated with age, *r* (106) = −0.40, *p* < 0.001, indicating a shorter sleep duration as chronological age increases, as expected (Table 1). The average total sleep per night was 9.39 h (Figure 1A).

The average total hours of sleep per night was significantly correlated with the total CSHQ, *r*(89) = −0.483, *p* < 0.001, and all CSHQ subscales except for Parasomnias (Table 1). A chi-square test found that children with less than 9 h of sleep were found to be significantly more likely to have clinical CSHQ scores: χ^2^(1) = 8.34, *p* = 0.004, and Cramer’s V = 0.30 (Figure 1B). The descriptive statistics of the CSHQ scores in this group are summarized in Table 4.

### 3.5. Anxiety Levels Based on SCAS Parental Scores

Descriptive statistics of the SCAS are summarized in Table 5. Neither the mean total SCAS score in the sample nor any sub-score showed elevated values.

**Figure 2:**
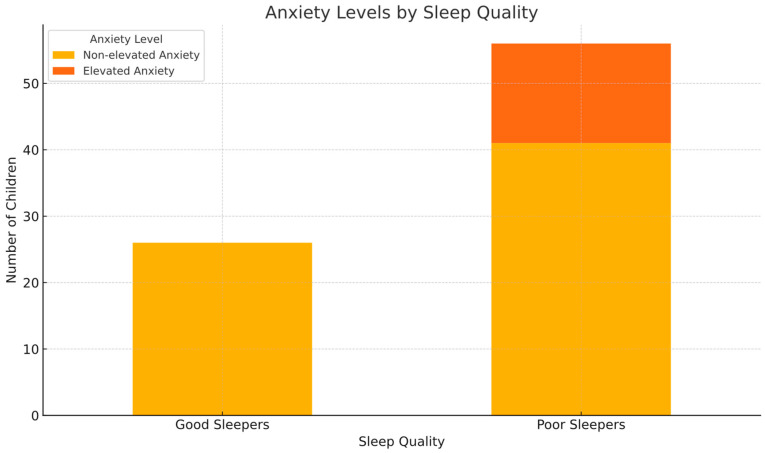
Observed frequencies in the Poor Sleepers group compared to the elevated anxiety group.

Children were categorized into elevated and non-elevated levels of anxiety according to the population norms for total SCAS scores, where 18.3% of the children in the sample (*n* = 15) showed elevated levels of anxiety (Figure 2). A chi-square test found that children with elevated anxiety were found to be significantly more likely to have clinical CSHQ scores: χ^2^(1) = 8.52, *p* = 0.004, and Cramer’s V = 0.32.

The total SCAS score was significantly correlated with the total CSHQ: *r* (80) = 0.433 and *p* < 0.001 (Table 1). The total SCAS score was also significantly correlated with five CSHQ subscales (Table 1): Sleep Anxiety (*r*(88) = 0.57, *p* < 0.001), Night Wakings (*r*(89) = 0.38, *p* < 0.001), Parasomnias (*r*(89) = 0.37, *p* < 0.001), Bedtime resistance, *r*(89) = 0.32, *p* = 0.002, and Sleep Duration (*r*(89) = 0.25, *p* = 0.017). The only item in the SCAS questionnaire that was significantly correlated with CSHQ Sleep Onset Delay was “I feel afraid I will make a fool of myself” (*r*(90) = 0.353, *p* < 0.001), whereas the following school anxiety-related items did not correlate: “I feel afraid if I have to talk in front of my class”, “I have trouble going to school in the mornings because I feel nervous or afraid”, “I worry that I will do badly at my school work” and “I feel scared when I have to take a test”.

## 4. Discussion

This study aimed to characterize sleep in children with dyslexia and its possible association with anxiety in a community sample. Overall, the results show that the majority of children in the dyslexia group had scores within the clinical sleep disturbances range. A significant correlation between reported sleep quality and anxiety symptoms in children was observed.

Children with dyslexia were reported to sleep 9.39 h on average, which is on the lower end of the recommended 9–12 h recommended for the younger age group [55]. This is in line with other studies in the literature report for both the sample of children with dyslexia and the sample of typically developing children, with results close to or below 9 h of sleep [21,23,25].

Sleep duration correlated with age, and it was an expected result that older children would sleep less than younger children. Moreover, sleep duration was correlated with total sleep problems, and those with less than the recommended sleep duration had higher CSHQ scores than those with sufficient sleep. However, almost 60% of children with enough sleep also reported sleep issues at the clinical level, indicating that parents who reported their child to sleep in sufficient amounts may still exhibit problematic sleep characteristics. This highlights the need for further research with objective measurements of sleep duration.

The current study suggests that children with dyslexia may be more likely to experience sleep disturbances than their typically developing peers. Sleep quality, as measured by the CSHQ, was reported to be poor in 66% of children with dyslexia, more than previous findings by Carotenuto and colleagues [16]. Children in the Poor Sleepers group showed clinical scores for the subscales of Sleep Onset Delay, Sleep Anxiety, and Daytime Sleepiness, but only Sleep Onset Delay was consistently reported at pathological levels in previous studies [10,16]. This is an important new finding that provides more detailed characteristics of sleep profiles in individuals with dyslexia.

Clinical levels of anxiety were only reported by 18% of parents. The total anxiety score was significantly correlated with Total Sleep Problems, Sleep Anxiety, Night Wakings, Parasomnias, and Bedtime Resistance, indicating a connection between anxiety and sleep disturbances, which is in line with the findings in previous studies [56]. Children with elevated anxiety were also more likely to have CSHQ scores within the clinical range, indicating an association between clinical sleep characteristics and anxiety symptoms, which is consistent with previous work demonstrating relationships between anxiety and sleep.

However, anxiety levels were not elevated, and in particular school-related anxiety items did not correlate with the main sleep problem: Sleep Onset Delay. However, we did not collect the data directly from the children but from their parents; hence, some of the issues related to anxiety might not be visible to the parents, and the sleep autonomy of older children might lead to underestimations of their sleep problems.

The present study is the first to evaluate sleep behavior in a large cohort of children with a diagnosis of dyslexia only. Given the high prevalence of comorbidities in neurodevelopmental disorders and the well-documented impact of ADHD [2] and autism [11,12,13] on sleep, the exclusion of participants with other suspected diagnoses strengthens the results on sleep characteristics in children with dyslexia. In addition, the recruitment of participants in the general dyslexia help network meant that the cohort was less biased towards sleep problems than in previous large studies when participants were recruited in clinical settings [10,19] and where sleep problems were potentially over-represented.

The findings highlight the need to explore the impact of well-established therapeutic options for sleep improvement in dyslexic populations, such as behavioral interventions and CBT-I. Furthermore, while better sleep is known to be associated with improvements in attention, working memory, and academic performance in typically developing children, the potential for similar effects needs to be explored in dyslexic populations.

The high prevalence of sleep disturbances in children with dyslexia raises important questions about the role of sleep in their cognitive and academic functioning. Although behavioral interventions [57] and CBT-I [58] are recognized as effective in typically developing populations, insights into their impact on dyslexic children remain limited. Given the established link between sleep and cognitive performance in non-dyslexic individuals [4], future research should examine whether similar interventions support cognitive functioning in children with dyslexia.

This study has a number of limitations, most notably the lack of a control group, meaning that the conclusions drawn are less reliable without a comparison. Also, in previous studies, the diagnosis of dyslexia in children was reported by participants and not independently verified. In addition, there may be a selection bias, as parents who turn to dyslexia self-help organizations might have children who are experiencing greater difficulties compared to those who do not seek support. Moreover, parental reports [59] provide a subjective view of the sleep behavior of their children; a comparison between parental reports and actigraphy results showed a high correlation with sleep schedule variables but lower associations with sleep quality measures [60]. In recent studies, CSHQ values have been raised for typically developing children; however, this may be due to the lack of excluding sleep-impacting conditions [53].

## 5. Conclusions

This is the first community study explicitly indicating pathological disturbances in sleep and an association with anxiety symptoms in children with dyslexia without other common comorbidities such as ADHD or autism. These findings are in line with the few previous studies conducted; therefore, given the high prevalence of dyslexia and the undisputed impact of sleep on cognitive functioning and learning, the further exploration of fine-grain sleep disturbances and mental health using objective sleep measures and self-reporting by children themselves is urgently needed. This research is important as it opens the potential for finding additional ways to support children with dyslexia to improve their cognitive performance and needs to be pursued alongside all current endeavors to improve the diagnostic criteria for dyslexia and important discussions on the effects of labeling. The possible underlying mechanisms of mental health factors, such as anxiety, but also neurobiological factors, such as HPA dysregulation, atypical cortisol levels, and possibly resulting circadian rhythm disorders, need to be considered. Such studies can provide valuable insights into developing interventions to improve sleep quality in children with dyslexia, addressing both physiological and behavioral aspects. This approach can support the creation of evidence-based interventions grounded in empirical findings.

## Figures and Tables

**Table 1:** Table of correlations among variables from the analysis of the sample.

	Age	CSHQ Total	CSHQ Bedtime Resistance	CSHQ Sleep Duration	CSHQ Daytime Sleepiness	CSHQ Night Wakings	CSHQ Parasomnias	CSHQ Sleep Onset	CSHQ Sleep Anxiety	CSHQ Sleep Disordered Breathing	SCAS Total	SCAS Separation Anxiety	SCAS Social Phobia	SCAS Obsessive Compulsive	SCAS Panic Agoraphobia	SCAS Physical Injury	SCAS General Anxiety
CSHQ Total	−0.14																
CSHQ Bedtime Resistance	−0.11	0.71 **															
CSHQ Sleep Duration	−0.03	0.61 **	0.47 **														
CSHQ Daytime Sleepiness	0.12	0.68 **	0.27 **	0.37 **													
CSHQ Night Wakings	−0.14	0.62 **	0.42 **	0.27 *	0.18 *												
CSHQ Parasomnias	−0.18	0.63 **	0.27 **	0.19 **	0.29 **	0.46 **											
CSHQ Sleep Onset Delay	−0.08	0.53 **	0.33 **	0.63 **	0.37 **	0.24 **	0.18 *										
CSHQ Sleep Anxiety	−0.18 *	0.70 **	0.78 **	0.37 **	0.22 *	0.44 **	0.40 **	0.27 **									
CSHQ Sleep Disordered Breathing	0.007	0.32 **	0.08	0.11	0.09	0.22 *	0.35 **	0.07	0.14								
SCAS Total	−0.06	0.43 **	0.32 **	0.25 *	0.12	0.38 **	0.37 **	0.19	0.57 **	0.12							
SCAS Separation Anxiety	−0.15	0.45 **	0.47 **	0.35 **	0.10	0.31 **	0.24 *	0.18	0.61 **	0.05	0.80 **						
SCAS Social Phobia	0.13	0.37 **	0.21 *	0.28 **	0.19	0.29 **	0.37 **	0.29 **	0.41 **	0.14	0.79 **	0.52 **					
SCAS Obsessive Compulsive	−0.07	0.25 *	0.16	0.12	0.07	0.28 **	0.24 *	0.12	0.29 **	0.13	0.73 **	0.43 **	0.47 **				
SCAS Panic Agoraphobia	0.01	0.35 **	0.19	0.15	0.05	0.41 **	0.37 **	0.15	0.43 **	0.12	0.90 **	0.61 **	0.66 **	0.70 **			
SCAS Physical Injury	−0.21 *	0.18	0.17	0.05	0.06	0.13	0.06	−0.07	0.41 **	0.06	0.45 **	0.38 **	0.21 *	0.18	0.29 **		
SCAS General Anxiety	−0.06	0.34 **	0.23 *	0.11	0.07	0.28 **	0.38 **	0.13	0.43 **	0.05	0.86 **	0.64 **	0.59 **	0.59 **	0.76 **	0.25 *	
Average Hours Of Sleep Per Night	−0.40 **	−0.48 **	−0.42 **	−0.59 **	−0.24 *	−0.36 **	−0.20	−0.58 **	−0.31 **	−0.20 *	−0.25 *	−0.25 *	−0.30 *	−0.15	−0.22	−0.01	−0.14

Note. *p* < 0.05 *, *p* < 0.01 **.

**Table 2:** Descriptive statistics of CSHQ for dyslexic individuals and the TD control group—clinical cut-off, means, and standard deviations.

	Clinic Cut-Off	All Dyslexic			Control Group (1)		
		*n*	Mean	SD	*n*	Mean	SD
Total CSHQ	41	116	**46.94**	9.04			
Bedtime resistance	9.43	129	8.13	2.94	382	7.06	1.89
Sleep Onset Delay	1.80	129	**1.85**	0.86	403	1.25	0.53
Sleep Duration	4.94	129	4.47	1.91	398	3.41	0.93
Sleep Anxiety	7.09	128	6.23	2.23	374	4.89	1.45
Night Waking	5.69	129	3.95	1.42	384	3.51	0.89
Parasomnias	11.22	122	9.39	1.94	371	8.11	1.25
Disordered Breathing	4.71	124	3.39	0.91	382	3.24	0.63
Daytime Sleepiness	11.99	127	10.87	3.42	381	2.80	9.64

(1) Values for the control group from the control sample, Owens [48]; bold—above clinical cut-off point.

**Table 3:** Descriptive statistics of the CSHQ for Poor Sleepers and Good Sleepers—clinical cut-off, means, and standard deviation.

	Clinic Cut-Off	Poor Sleepers Total CSHQ > 41			Good Sleepers Total CSHQ ≤ 41		
Total CSHQ	41	40	**51.86**	7.17	76	37.60	2.41
Bedtime resistance	9.43	40	9.13	3.21	76	6.10	0.30
Sleep Onset Delay	1.80	40	**2.03**	0.85	76	1.45	0.71
Sleep Duration	4.94	40	4.88	2.01	76	3.40	0.84
Sleep Anxiety	7.09	40	**7.16**	2.19	76	4.40	0.81
Night Wakings	5.69	40	4.39	1.59	76	3.10	0.38
Parasomnias	11.22	40	10.05	1.96	76	8.13	1.09
Disordered Breathing	4.71	40	3.57	1.10	76	3.10	0.30
Daytime Sleepiness	11.99	40	**12.12**	3.18	76	7.98	1.85

Bold—above the clinical cut-off point.

**Table 4:** Descriptive statistics for the CSHQ above and those below the reported 9 h of sleep needed—clinical cut-off, means, and standard deviation.

	Clinic Cut-Off	<9 h Sleep			≥9 h Sleep		
Total CSHQ	41	34	**51.32**	8.68	73	**45.00**	8.03
Bedtime resistance	9.43	37	9.43	3.44	78	7.50	2.69
Sleep Onset Delay	1.80	37	**2.38**	0.76	78	1.60	0.76
Sleep Duration	4.94	37	**5.84**	2.17	78	3.80	1.58
Sleep Anxiety	7.09	37	6.78	2.49	78	5.86	2.04
Night Wakings	5.69	37	4.16	1.28	78	3.69	1.17
Parasomnias	11.22	35	9.54	1.58	76	9.21	1.73
Disordered Breathing	4.71	36	2.49	0.88	77	3.30	0.72
Daytime Sleepiness	11.99	37	11.92	3.74	78	10.34	3.17

Bold—above the clinical cut-off point.

**Table 5:** Descriptive statistics of SCAS scores in the sample.

	*n*	Mean	SD
Total SCAS	91	28.78	15.44
Separation Anxiety	92	5.33	3.82
Social Phobia	92	8.00	3.81
Obsessive Compulsive	92	2.71	2.51
Panic Agoraphobia	91	3.42	3.92
Physical Injury	92	3.40	2.33
General Anxiety	92	5.97	3.42

## Data Availability

The datasets presented in this article are not readily available due to technical limitations. Requests to access the datasets should be directed to the authors.
